# Evaluating the impact of lactase supplementation on infant colic: Study protocol for a systematic review of randomized controlled trials

**DOI:** 10.1002/jpr3.12024

**Published:** 2024-01-23

**Authors:** Anna Kozłowska‐Jalowska, Agata Stróżyk, Andrea Horvath, Hania Szajewska

**Affiliations:** ^1^ Department of Pediatrics The Medical University of Warsaw Warsaw Poland

**Keywords:** children, excessive crying, lactose intolerance

## Abstract

Infant colic is a common functional gastrointestinal disorder that affects infants during their first months of life. The etiology of this condition remains unclear. However, some studies suggest lactase deficiency may be a contributing factor. Currently, the evidence on dietary treatment and lactase supplementation for management of infant colic is limited. We aim to systematically review evidence on the efficacy and safety of using a lactase supplementation for managing infant colic. The Cochrane Central Register of Controlled Trials (CENTRAL, the Cochrane Library), MEDLINE, and EMBASE will be searched to identify randomized controlled trials comparing oral lactase supplementation with placebo or no intervention in infants aged less than 6‐month‐old with infant colic using any recognized definition. The risk of bias will be assessed using the second version of the Cochrane Collaboration's risk‐of‐bias tool. The main outcome will be the number of responders in each group after treatment, defined as infants who experienced a decrease in daily crying as reported by the study authors. Additional outcomes will include the duration and frequency of crying episodes, infant sleep duration, parental satisfaction, discomfort of infants, number of hospital admissions, family quality of life, and adverse events during the intervention. The study findings will be published in a peer‐reviewed journal and will be submitted to relevant conferences.

## Abstract (highlights)

**What is Known:**

- Although infant colic is a common problem during first months of life, there is no well‐established treatment.
- Evidence on use of lactase supplementation in treatment of infant colic is limited.

**What is New:**

- This systematic review of rigorous methodological design will evaluate the evidence on the efficacy and safety of using lactase supplementation for the management of infant colic.
- Quality assessment of randomized controlled trials considering lactase supplementation in infant colic will be performed.

**Translational Impact:**

- The findings of this systematic review may guide further studies and the development of clinical practice guidelines.

## 1 INTRODUCTION

Infant colic is a common functional gastrointestinal disorder that affects children during their first months of life.^1^ The main symptoms are recurrent and prolonged periods of infant crying, fussing, or irritability that occur without obvious cause and cannot be prevented or resolved by caregivers.^2^ The diagnostic criteria of infant colic have changed over the years (Table 1). Currently, the Rome IV Criteria^5^ are recommended to recognize infant colic. According to a 2017 systematic review the prevalence of infant colic varies depending on the infant's age, with the highest prevalence of up to 25.1% occurring in infants aged 5 to 6 weeks, and the lowest prevalence at 10 to 12 weeks of age (0.6%).^6^ Although infant colic is not considered a serious clinical condition, it may lead to parental distress, child abuse, and early breastfeeding cessation.^7^ Moreover, recent studies suggest that infant colic also may lead to long‐term consequences such as functional gastrointestinal disorders, headache of the migraine type, and behavioral problems.^7^, ^8^ The etiology of the condition remains unclear. However, some studies suggest that infant colic may be caused by lactase deficiency. A 2018 Cochrane systematic review considering dietary modification in infant colic treatment found that current evidence is insufficient to make any conclusion on the effectiveness of lactase supplementation in the management infant colic⁠.^1^, ^2^, ^7^ However, additional trials have been published in the last 5 years.^9^, ^10^ We aim to systematically review evidence on the efficacy and safety of using lactase supplementation for the management of infant colic.

**Table 1:** Summary of the diagnostic criteria of infant colic.

Criterion	Year	Definition
Wessel criteria^3^	1954	Paroxysms of crying episodes, lasting for more than 3 h per day, for 3 or more days per week, and for 3 or more weeks
Rome III^4^	2006	Diagnostic criteria must include all of the following in infants from birth to 4‐month of age: Paroxysms of irritability, fussing or crying that starts and stops without obvious cause; Episodes lasting 3 or more h/day and occurring at least 3 day/week for at least 1 week; No failure to thrive.
Rome IV^5^	2016	Diagnostic criteria for clinical purpose must include all of the following in infants from birth to 5‐month of age when symptoms start and stop: Recurrent and prolonged periods of fussing/crying/irritability; which cannot be prevented or resolved by care‐givers; No evidence of failure to thrive, fever or illness. Diagnostic criteria for clinical research must meet the preceding diagnostic criteria and also include both of the following: Caregiver reports infant has cried or fussed for 3 or more h/day during 3 or more days in 7 days in a telephone or face‐to‐face screening interview with a researcher or clinician; Total 24‐h crying plus fussing in the selected group of infants is confirmed to be 3 h or more when measured by at least one, prospectively kept, 24‐h behavior diary.

## 2 METHODS

This protocol follows the Preferred Reporting Items for Systematic review and Meta‐Analysis Protocols (PRISMA‐P) guidelines for systematic review protocols (for checklist, see Supplemental Digital Content 1). The Cochrane Handbook for Systematic Review of Intervention Version 6.3.^11^ ⁠ and PRISMA guidelines for undertaking and reporting the results of a systematic review and meta‐analysis^12^ will be followed at each stage of this review⁠. The study protocol has been registered with PROSPERO under the registration number CRD42023441112.

### 2.1 Eligibility criteria

#### 2.1.1 Type of studies

Only randomized controlled trials (RCTs) will be included (regardless of language, study duration, settings, length of follow‐up, and country incomes). All excluded trials with reasons for exclusion will be reported in the “Characteristics of excluded trials” table. Ongoing studies will be excluded; however, they will be reported in the “Characteristics of ongoing studies” table.

#### 2.1.2 Type of population

Studies involving infants aged less than 6‐month‐old diagnosed with infant colic by any recognized criteria will be included. Both breastfed and/or formula‐fed infants will be eligible for inclusion.

#### 2.1.3 Type of intervention and comparator

Studies using oral lactase supplementation compared with placebo or no intervention will be eligible for inclusion.

#### 2.1.4 Type of outcomes measures

The main outcome measure will be the number of responders in each group after treatment. Responders are defined as those infants who experienced a decrease in daily crying, as reported by the study authors.

Additional outcomes will include:

1. Duration of crying (minutes per day) during the intervention, measured with any method used by the investigators;
2. Frequency of crying episodes per day during the intervention;
3. Infant sleep duration (minutes per day) during the intervention;
4. Satisfaction in terms of positive changes in child's mood during the intervention measured with any method used by the investigators (i.e., Likert or numeric rating scale);
5. Discomfort of infants during the intervention measured with any method used by the investigators;
6. Number of hospital admissions during the intervention;
7. Family quality of life during the intervention measured with any method used by the investigators;
8. Adverse events during the intervention.

Outcomes to be assessed in this review are based on core outcome set for infant colic.^13^

#### 2.1.5 Information sources

The Cochrane Central Register of Controlled Trials (CENTRAL, the Cochrane Library), MEDLINE (PubMed), and EMBASE will be searched for studies from 1947 to June 2023 using a prespecified search strategy. Searches will be re‐run just before the final analyses, and any further studies identified will be retrieved for inclusion. Letters to the editor, abstracts, and proceedings from scientific meetings will be excluded unless a full set of data is available from the authors. Manual searches of the reference lists of the included studies and key review articles, as well as previously published systematic reviews/meta‐analyses that assessed the effects of lactase enzyme for the management of infant colic also will be searched. The International Clinical Trial Registry Platform (ICTRP, https://trialsearch.who.int/) also will be searched manually to identify any unpublished or ongoing trials.

### 2.2 Study records

#### 2.2.1 Study selection process

First, two reviewers (A. K. J., A. S.) will independently screen the titles, abstracts, and keywords of every study identified with the search strategy, with the use of EndNote X9 Computer program (Version 9.3.3. Philadelphia, The Clarivate Analytics, 2020). Then, full texts of publications of potentially relevant trials will be retrieved and independently assessed against eligibility criteria by the reviewers. Trials that fulfill eligibility criteria will be included. Any discrepancies will be resolved through discussion; if required, another review author will be consulted (A. H. or H. S.).

#### 2.2.2 Data collection and management process

Two reviewers (A. K. J., A. S.) will use a standardized form to extract independently data from included trials. Reviewers will attempt to contact the authors of the original study whenever reported data are insufficient for planned analysis. The process of selection will be presented as the PRISMA flow diagram. All excluded trials with reasons for exclusion will be reported in the “Characteristics of excluded trials” table.

#### 2.2.3 Data items

Two reviewers (A. K. J., A. S.), using a standardized form, will extract independently the following data from the included trials:

1. Methods (study design, study duration, year of publication, authors, country);
2. Participants (number, sex, age, infant colic diagnostic criteria, type of feeding, other population characteristics);
3. Description of intervention (i.e., lactase dose, frequency and type of administration, duration) and number of participants in the intervention group;
4. Description of comparator (placebo or no intervention) and number of participants in the control group;
5. Outcomes (data for main and additional outcomes with timeframe of evaluation);
6. Sample size calculation;
7. Funding and/or conflict of interest.

Any disagreements will be resolved by discussion; if required, another reviewer will be consulted (A. H. or H. S.). The data items extracted from the included trials will be reported in a “Characteristics of included studies” table.

#### 2.2.4 Risk of bias in included studies

The risk of bias will be assessed independently by two reviewers (A. K. J., A. S.). The second version of the Cochrane Collaboration's risk‐of‐bias tool for randomized trials (RoB 2)^14^ ⁠ will be used. Assessments will be made in five bias domains: bias introduced during the randomization process, bias caused by deviations from intended interventions, bias due to missing outcome data, bias in the measurement of the outcome, and bias in the selection of the reported result. Based on this assessment, the overall bias will be judged to determine whether the study has a low risk of bias, a high risk of bias or raises some concerns. The assessment will be done at the study level. Possible disagreements between each of the reviewers' judgements will be resolved by discussion; if required, another reviewer will be consulted (A. H. or H. S.).

#### 2.2.5 Measure of treatment effect

All outcomes will be reported as the difference between study groups for each study point reported by the authors. We will report a risk ratio (RR) between the experimental and control groups with 95% confidence intervals (CIs) for dichotomous outcomes. If feasible, the number needed to treat (NNT) will be calculated. The mean difference (MD) between the treatment and control groups will be selected to represent the difference in continuous outcomes (with 95% CIs).

#### 2.2.6 Data synthesis

Data will be independently extracted by two of the reviewers (A. K. J., A. S.). Then it will be entered into the Review Manager (RevMan) computer program (Version 5.4. The Cochrane Collaboration, 2020). All analyses will be based on the random‐effect model. In case of substantial heterogeneity of the analyzed population, intervention, comparators, and outcomes, the results of the trials will not be pooled in a meta‐analysis but will be described in narrative form.

#### 2.2.7 Heterogeneity

The heterogeneity of the trials will be determined using the estimation of inconsistency (*I*², where ≥50% is defined as “substantial heterogeneity” in accordance with the Cochrane Handbook), as it seems to be more reliable than the *χ* ^2^ test.

#### 2.2.8 Subgroup analysis

The analysis of subgroups based on type of feeding (breastfed/formula‐fed), infant colic definition criteria (Wessel/Rome IV criteria/other), and trial quality (low/high risk of bias) will be conducted, if feasible. Anticipated differences between prespecified subgroups are based on the results of previous systematic reviews on the management of infant colic (i.e., different effects *of Lactobacillus reuterii* DSM 17938 treatment in breast‐fed and formula‐fed infants⁠).^15^ For the subgroup analysis the test for subgroup differences in RevMan will be used and interpreted following the Cochrane Handbook.^11^ ⁠The random effect model will be used for analysis within each subgroup.

#### 2.2.9 Metabias

When at least 10 RCTs available, publication bias will be assessed using the funnel plot proposed by Egger et al.^16^ A *p* < 0.05 implicates publication bias.

#### 2.2.10 Confidence in cumulative evidence

If feasible, the certainty of evidence will be assessed using the grading of recommendations, assessment, development, and evaluation (GRADE) methodology.^17^

#### 2.2.11 Dissemination

The findings of this review will be published in a peer‐reviewed journal in accordance with PRISMA. Abstracts will be submitted to relevant national and international conferences.

## 3 CONCLUSION

Currently, there is a lack of systematic reviews assessing the efficacy and safety of using oral lactase supplementation for the management of infant colic. Therefore, our review aims to fill this knowledge gap. However, it is important to note that this review may be constrained by the quality and number of trials that meet the eligibility criteria. Nevertheless, the findings from our systematic review hold the potential to inform and guide future studies and the development of clinical practice guidelines in this area.

## CONFLICT OF INTEREST STATEMENT

The authors declare no conflict of interest.
